# CD44 Expression Predicts Prognosis of Ovarian Cancer Patients Through Promoting Epithelial-Mesenchymal Transition (EMT) by Regulating Snail, ZEB1, and Caveolin-1

**DOI:** 10.3389/fonc.2019.00802

**Published:** 2019-08-21

**Authors:** Jiayi Zhou, Yan Du, Yiling Lu, Baoxin Luan, Congjian Xu, Yinhua Yu, Hongbo Zhao

**Affiliations:** ^1^Obstetrics and Gynecology Hospital, Fudan University, Shanghai, China; ^2^Department of Obstetrics and Gynecology of Shanghai Medical School, Fudan University, Shanghai, China; ^3^Shanghai Key Laboratory of Female Reproductive Endocrine Related Diseases, Shanghai, China; ^4^Department of Systems Biology, University of Texas MD Anderson Cancer Center, Houston, TX, United States

**Keywords:** CD44, expression, ovarian cancer, epithelial-to-mesenchymal transition, prognosis, survival

## Abstract

**Objectives:** CD44, a transmembrane glycoprotein, is involved in the generation of a stem cell niche and maintaining stem cell quiescence. The aim of this study was to evaluate its contribution to ovarian cancer prognosis and progression, as well as explore the possible mechanisms.

**Materials and Methods:** The expression of CD44 in tissue microarray of 90 ovarian cancer patients was detected by immunohistochemistry. Kaplan-Meier method and Cox proportional hazard model were used to evaluate the factors associated with 5-year overall survival and disease-free survival. CD44 was knocked down by small interfering RNA, the expression of Snail, ZEB1, and Caveolin-1 in a stable Snail-expressing ovarian cancer cell line HO8910PM-Snail (HOPM-Snail) and its control cell line HO8910PM-vector (HOPM) was detected by western blotting analysis. Cell clone formation, migration, and invasion of HOPM-Snail and HOPM cells with CD44 silencing were examined by 3-D culture assay, wound healing assay, and transwell assay, respectively.

**Results:** Over-expression of CD44 was associated with advanced histological grade (*p* = 0.014) and FIGO stage (*p* = 0.001). Multivariate analysis showed that CD44 expression was an independent prognostic factor to predict both overall survival (*p* = 0.004) and disease-free survival (*p* = 0.025) of ovarian cancer patients. Down-regulation of CD44 expression by small silencing RNA abrogated both basal Snail expression and TGF-β1-induced Snail expression in HOPM and HOPM-Snail cells. In addition, CD44 knockdown caused a decrease in ZEB1 expression. RPPA data indicated that Caveolin-1 may be another regulative target of CD44, and western blotting analysis confirmed that CD44 knockdown caused an increase in Caveolin-1 expression. However, there was no noticeable reciprocal regulation among ZEB1, Caveolin-1, and Snail. Moreover, CD44 knockdown caused a decrease in cell clone formation, migration, and invasion of HOPM and HOPM-Snail cells.

**Conclusions:** As both Snail and ZEB1 are crucial inducers of epithelial-to-mesenchymal transition (EMT), our data suggested that CD44 may be crucial for the EMT process of ovarian cancer. Therefore, CD44 may be a potential prognostic marker as well as treatment target for ovarian cancer.

## Introduction

Ovarian cancer is one of the most common gynecologic cancer and a fatal malignancy in women worldwide (1). There were estimated 295,414 new cases of and 184,799 deaths caused by ovarian cancer in 2018 (1). With an aging population, ovarian cancer is and will continue to be a huge health burden in China (2). Epidemiological studies have identified several risk factors including menstrual and reproductive factors, obesity, hormone therapy, personal history of breast cancer, family history, and genetic mutations (*BRCA1* and *BRCA2* mutations) (3, 4). However, the etiology of ovarian cancer is still unclear. Despite treatment advancement in recent decades, the prognosis of ovarian cancer patients remains poor, with a reported 5-year survival rate of 45% in the United States (5). Therefore, it is of vital importance to identify the predictive markers of recurrence risk and survival, as well as therapeutic targets.

CD44, as a transmembrane glycoprotein, is involved in the generation and maintenance of the stem cell niche and self-renewal potential (6). It has been well-documented that high expression of CD44 predicts poor prognosis of various tumors including breast, brain, colon, pancreatic, and gastric tumors, indicating that CD44 may be a valuable prognostic marker and therapeutic target for cancers (7). Although the association of CD44 expression with the survival of ovarian cancer patients has been widely investigated, the role of CD44 in the prognosis of ovarian cancer remains controversial (8–18). Some studies found that increased expression of CD44 closely correlated with poor prognosis of ovarian cancer (8–12). On the contrary, other studies reported that CD44 was not an independent predictor of survival and prognosis (13–18).

Ovarian cancer frequently consists of heterogeneous subpopulations. Among these cell populations, cancer stem cells (CSCs) have been widely accepted to endow ovarian cancer with tumor initiation and self-renewal potential (19, 20). CD44, as the most frequently reported CSC marker, is widely used to distinguish CSCs from other populations of cancer cells (21–23). CD44, as well as other CSC markers endoglin (CD105) and CD106 has been proved to be highly expressed in chemo-resistant ovarian cancer cells and in advanced-stage epithelial ovarian cancer tissues, suggesting CD44 may accelerate the progression of ovarian cancer by modulating the properties of CSCs (24).

Epithelial-to-mesenchymal transition (EMT), which enables the invasion of epithelial carcinoma cells to the underlying stroma, is a critical pathophysiological process in epithelial cancer (25). EMT is induced and maintained by critical genes including N-cadherin, Snail, Slug, Twist, Vimentin, and Zinc finger E-box-binding homobox 1 (ZEB1) (26–28). Previous data have shown that CD44 over-expression caused a significant up-regulation of the mesenchymal markers N-cadherin and Vimentin with a concomitant down-regulation of the epithelial markers E-cadherin and Claudin 7 in PA1 and SKOV3 ovarian cancer cells, indicating the possible involvement of CD44 in EMT (29). However, the exact role of CD44 in EMT in ovarian cancer remains elusive and requires further investigation.

In the present study, we systematically evaluated the prognostic value of CD44 in ovarian cancer patients, and explored the modulation of CD44 on EMT in ovarian cancer cell lines. Our study has proposed a new mechanism by which CD44 induces the EMT progress *via* ZEB1 and Snail in ovarian cancer. Therefore, CD44 may be a potential target for ovarian cancer not only due to its effect on stem cell properties, but also due to its pivotal role in EMT.

## Materials and Methods

### Study Patients

This is a retrospective cohort study. We included patients with newly diagnosed sporadic epithelial ovarian cancer at the Obstetrics and Gynecology Hospital of Fudan University in Shanghai, China from May 2006 to November 2008. Specimens were collected by the Tissue Bank of the hospital. Inclusion Criteria were: (1) patients with newly diagnosed sporadic epithelial ovarian cancer, (2) written informed consent was obtained. Exclusion Criteria were: (1) patients with history of other malignant tumors, (2) did not receive surgical treatment at our hospital. The study protocol was approved by the Institutional Review Board (IRB) of the hospital (Reference number: 2018-24; Date of approval: 2 April 2018). Each participant gave written informed consent. Clinical and histopathologic information was retrospectively collected from individual's medical records. The following variables were extracted: age at diagnosis (years), menopause (yes or no), laterality (right side, left side, or bilateral), behavior (borderline or invasive), histological subtype (serous or non-serous), histological grade (G1, G2, or G3), Federation of Gynecologists and Obstetricians (FIGO) stage (I, II, III, or IV), intravascular tumor thrombus (yes or no), serum CA125 (<35 or ≥35 U/mL), serum CA19-9 (<37 or ≥37 U/mL), serum CEA (<5 or ≥5 ng/mL), and chemotherapy (yes or no).

### Follow-Up

Follow-up was conducted at the dedicated unit in our hospital according to standard epidemiologic protocol (30). Follow-up started 6 months after the surgery and was performed by examinations every 3 months on an outpatient bases and/or by telephone calls. Follow-up information including overall survival (OS, in months), and disease-free survival (DFS, in months) was linked to the clinicopathological database using the unique patient ID number. All identifiable data was removed once the dataset was constructed to protect patient's privacy.

### Immunohistochemistry and Scoring

The ovarian cancer tissues were obtained from the Tissue Bank of Obstetrics and Gynecology Hospital of Fudan University. The construction of tissue microarray was previously described (30). Ovarian cancer tissues were fixed in 4% paraformaldehyde at 4°C overnight, dehydrated by graded ethanol solutions and embedded in paraffin. The tissue sections (5-μm) were deparaffinized and subjected to heat-induced antigen retrieval. The endogenous peroxidase activity was quenched after incubation in 3% hydrogen peroxide for 10 min. The sections were incubated with 10% goat serum for 30 min to block non-specific binding sites and then incubated with the primary antibodies (1:100) at 4°C overnight followed by secondary antibody (1:100) for 1 h at 37°C. Bound antibody was then visualized using the EnVision™ Detection Systems (Dako, Glostrup, Denmark). The expression of CD44 was examined and evaluated using immunohistochemistry according to our previous protocol (30). The expression was independently reviewed by two observers (YD and HZ). The immunostaining score was incorporated both staining intensity (0 = absent, 1 = weak, 2 = moderate, 3 = strong) and percentage of positive cells (0 = 0%, 1 = 1–25%, 2 = 26–50%, 3 = 51–75%, 4 = 76–100% of cells). The immunostaining score was calculated based on the proportion of stained tumor cells: 0–10% as negative (–), 11–25% as slightly positive (+), 26–50% as moderately positive (++), and 51–100% as strongly positive (+++). Patients with – and + expression was combined as the lower expression group, and patients with ++ and +++ expression was combined as the higher expression group for analyses (30).

### Reagents and Antibodies

Monoclonal antibodies to CD44, ZEB1, Caveolin-1, and GAPDH were obtained from Cell Signaling Technology (San Diego, CA, USA). CD44 construct was obtained from Asia-Vector Biotechnology (Shanghai, China). Secondary antibodies conjugated with HRP were obtained from Jackson ImmunoResearch Laboratories (West Grove, PA, USA). CD44, ZEB1, Caveolin-1 small interfering RNAs (siRNAs) were synthesized by RioBio Co. (Guangzhou, China). The target sequences of siRNA are indicated in Table S1.

### Cell Culture

HO8910PM-Snail (HOPM-Snail), a stable Snail-expressing cell line, and its control cell line HO8910PM-vector (HOPM) were generated as described previously (31). All the cells were cultured in 1640 complete medium supplemented with 10% FBS and 400 μg/ml of G418 in 5% CO_2_ at 37°C.

### Transfection

Cells were seeded in 6-well plates at a density of 10^5^/well, and transfected with CD44 siRNA or scrambled siRNA using Lipo2000 reagent according to the manufacturer's instructions. At 48 h post-transfection, the cell pellets were harvested for western blotting, cell migration, and invasion assay. The transfection accuracy was evaluated by detecting the expression of target genes using western blotting analysis at 48 h after transfection.

### Western Blotting

Ovarian cancer cells were lysed in 1 × SDS lysis buffer (50 mM Tris-HCl, pH 6.8, 2% SDS, 10% glycerol, 1 mM PMSF, and 1 mM Na_3_VO4) and performed as previously described (32). An equal amount of total protein from various cell lysates was loaded on a SDS-PAGE gel and transferred to PVDF membrane (Millipore Corporation, USA). The membrane was blocked with 5% BSA in PBS (containing 0.05% Tween 20) and then incubated with specific primary antibodies and followed by incubation with HRP-conjugated secondary antibodies (Jackson ImmunoResearch Laboratories, West Grove, PA, USA). The protein bands of interest were visualized by fluorography using an enhanced chemiluminescence system (Thermo Fisher Scientific, USA).

### Cell Invasion

Cell invasion were evaluated using transwell assay. Cells grown to 60–70% confluence were transfected with CD44 siRNA. At 48 h post-transfection, cells were harvested by trypisinzation, pelleted by centrifugation, washed twice with PBS and resuspended in 0.1% fetal bovine serum in 1640 medium at a density of 5 × 10^5^ cells/ml. 3 × 10^4^ cells were added in the upper chamber of an insert (pore size, 8 μm, Costar Corporation). The lower chamber was filled with 1640 medium containing 10% fetal bovine serum as an inducer. For invasion assay, 5 × 10^4^ of cells were placed into the upper chamber precoated with Matrigel (BD Biosciences, Bedford, MA, USA). After 48 h incubation and removal of the cells on the upper chamber of the filters with a cotton swab, the cells that migrated to the lower surface of the filters were fixed in 4% paraformaldehyde and stained with crystal violet. Migrating cells were monitored with a LEICA microscope (Olympus IX71, Japan). Five visual fields of each insert were randomly chosen and the number of cells that migrated to the lower surface was counted for each well. The assays were performed in triplicate.

### Wound Healing Assay

Cells were seeded in 6-well plates and transfected with CD44 siRNA, and grown to confluence. A linear wound was generated in the cell layer by scratching with sterile 200 μL pipette tips. Cellular debris was removed by washing with medium. From each of these scratches, three representative images of the scratched areas were photographed at 48 h to analyze the migration capacity. The experiments were performed in triplicate.

### 3D-matrigel Culture

The 3D matrigel-based culture system was performed according to the manufacture's instruction. Briefly, pre-cooled 8-well chamber slide was coated by matrigel (80–100 μl/well) at 37°C for 15 min. 5 × 10^4^/ml cells in 4% matrigel were seeded in 6-well plate and maintained in 37°C. The medium were changed every 3–4 days with 2% matrigel. Cell clones were monitored with a LEICA microscope (Olympus IX71, Japan).

### Reverse Phase Protein Array (RPPA) Analysis

HOPM and HOPM-Snail cells were transfected with CD44 siRNA and then subjected to RPPA analysis at the University of Texas, M.D. Anderson Cancer Center RPPA Core Facility. Briefly, cells were grown in 6-well plates at a density of 10^5^/well. At 48 h post-transfection, cells were lysed in lysis buffer (1% Triton X-100, 50 mM HEPES, pH 7.4, 150 mM NaCl, 1.5 mM MgCl_2_, 1 mM EGTA, 100 mM NaF, 10 mM Na_4_P_2_O_7_·10H_2_O, 1 mM Na_3_VO_4_, 10% glycerol, containing freshly added protease and phosphatase inhibitors). The cell lysates were centrifuged and collected to determine protein concentration by BCA. Cell lysates were boiled for 5 min, and stored in −80°C until sample submission.

### Statistical Analysis

Chi-square test or Fisher's exact test was used to determine the differences of clinicopathogical variables between high- and low-expression subgroups. Kaplan–Meier method was used to estimate OS and DFS, and log-rank test was used to compare the curves of different expression groups. Cox proportional hazards models were performed to estimate the survival distributions, and to calculate hazard ratios (HRs) and corresponding 95% confidence intervals (95% CIs). Variables that reach the statistical significance level in the univariate model were included in the multivariate analysis. For cell line studies, results were expressed as mean ± standard error of the mean (SEM), and were evaluated using one-way ANOVA followed by the Dunnett test. All significance tests were two sided; *p*-value of <0.05 was considered as statistically significant. Data analyses were performed by STATA version 15 (StataCorp LLC, College Station, TX, USA).

## Results

### Baseline Characteristics of the Study Population

A total of 90 ovarian cancer patients were included in the current study. The clinicopahtological characteristics of the study population are presented in Table 1. The average age of these patients was 51.2 ± 9.5 years old. There were more patients with serous ovarian cancer (*n* = 59, 65.6%). More than half of the patients were diagnosed at advanced stage (FIGO stages III-IV, *n* = 49, 54.5%).

**Table 1:** Clinicopathological characteristics of study patients (*n* = 90).

**Variables**	***N***	**%**
Age at diagnosis		
Median (range) [years]	51	26–74
Age		
≤ 50 years	42	46.7
>50 years	48	53.3
Overall survival		
Median (range) [months]	74.58	1.60-103.13
Number of deaths	43	47.8
Disease-free survival		
Median (range) [months]	67.65	0.80-103.13
Number of recurrences	47	52.2
Menopause		
Yes	45	50.0
No	45	50.0
Laterality		
Right side	24	26.7
Left side	18	20.0
Bilateral	48	53.3
Histological subtype		
Serous	59	65.6
Non-serous	31	34.4
Histological grade		
G1	8	8.9
G2	21	23.3
G3	37	41.1
Missing	24	26.7
FIGO		
I	28	31.1
II	13	14.4
III	44	48.9
IV	5	5.6
Intravascular tumor thrombus		
Yes	16	17.8
No	71	78.9
Missing	3	3.3
Serum CA125		
<35 U/mL	12	13.3
≥35 U/mL	64	71.1
Missing	14	15.6
Serum CA19-9		
<37 U/mL	39	43.3
≥37 U/mL	17	18.9
Missing	34	37.8
Serum CEA		
<5 ng/mL	60	66.7
≥5 ng/mL	2	2.2
Missing	28	31.1
Chemotherapy		
Yes	86	95.6
Missing	4	4.4
CD44		
–	28	31.1
+	13	14.4
++	44	48.9
+++	5	5.6

*CA 125, cancer antigen 125; CA 19-9, cancer antigen 19-9; CEA, carcinoembryonic antigen; FIGO, Federation of Gynecologists and Obstetricians*.

### Expression of CD44 in Ovarian Cancer Tissues

**Figure 1:**
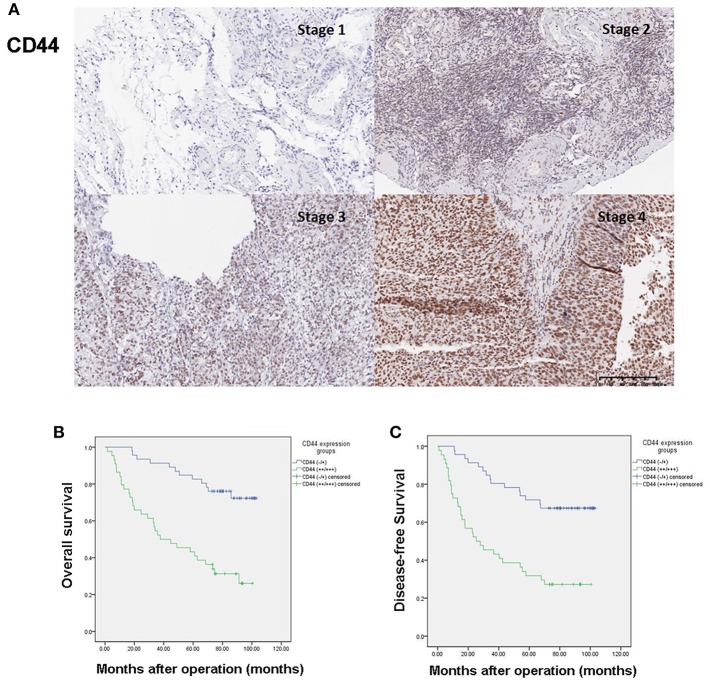
High expression of CD44 predicts poor prognosis in ovarian cancer patients. **(A)** Representative immunostaining of CD44 expression at different FIGO stages of ovarian cancer. **(B)** High expression of CD44 is associated with poor overall survival of ovarian cancer patients. **(C)** High expression of CD44 is associated with poor disease-free survival of ovarian cancer patients.

The immunohistochemistry data showed that CD44 was almost undetectable in healthy ovarian tissues (Figure S1) while mainly located in the nuclei of ovarian cancer tissue. Figure 1A shows the representative immunostaining of CD44 at different FIGO stages of ovarian cancer. The expression of CD44 increased with the advancement of FIGO stage. Table 2 presents the association between CD44 expression and clinicopathological characteristics. High expression of CD44 was associated with higher histological grade (*p* = 0.014), and more advanced FIGO stage (*p* = 0.001). There was no significant association between CD44 expression and any other clinicopahtological factors.

**Table 2:** Correlation of clinicopathological characteristics with CD44 expression in 90 ovarian cancer patients.

**Characteristics**	**CD44**		***p*-value**
	**–/+ (*n* = 46)**	**++/+++ (*n* = 44)**	
Age			0.844
≤ 50	21	21	
>50	25	23	
Menopause			0.673
Yes	22	23	
No	24	21	
Laterality			0.142*
Right side	11	13	
Left side	13	5	
Bilateral	22	26	
Histological subtype			0.065
Serous	26	33	
Non-serous	20	11	
Histological grade			**0.014**
G1/G2	19	10	
G3	13	24	
FIGO stage			**0.001**
I/II	29	12	
III/IV	17	32	
Intravascular tumor thrombus			0.615
No	35	36	
Yes	9	7	
Serum CA125 [U/mL]			0.059
<35 U/mL	9	3	
≥35 U/mL	27	35	
Serum CA19-9 [U/mL]			0.771
<37	20	19	
≥37	8	9	
Serum CEA [ng/mL]			0.184*
<5	29	31	
≥5	0	2	

* *Fisher's exact test. Bold values indicate p-value < 0.05*.

### High Expression of CD44 Predicts Poor Prognosis in Ovarian Cancer Patients

The median post-operative follow-up time was 74.58 months (range: 1.60–103.13 months). During the time period, 43 patients died, 4 relapsed but were still alive at the end of the study. Kaplan–Meier curves of CD44 expression and OS and DFS are presented in Figures 1B,C. High expression of CD44 significantly associated with both worse OS and DFS (Log-rank test *p* < 0.0001 for both). Univariate analysis results showed that advanced FIGO stage (III/IV vs. I/II, HR = 9.20, 95% CI = 3.86–21.96, *p* < 0.0001), intravascular tumor thrombus (Yes vs. No, HR = 2.08, 95% CI = 1.04–4.16, *p* = 0.039), elevated CA125 level (≥35 U/mL vs. <35 U/mL, HR = 4.62, 95% CI = 1.11–19.26, *p* = 0.035), and high expression of CD44 (+++/++ vs. +/–, HR = 4.30, 95% CI = 2.20–8.40, *p* < 0.0001) were associated with worse OS; while higher histological subtype (serous vs. non-serous, HR = 1.99, 95% CI = 1.01–3.92, *p* = 0.046), advanced FIGO stage (III/IV vs. I/II, HR = 11.36, 95% CI = 4.78–27.03, *p* < 0.0001), elevated CA125 level (≥35 U/mL vs. <35 U/mL, HR = 4.93, 95% CI = 1.19–20.44, *p* = 0.028), and high expression of CD44 (+++/++ vs. +/–, HR = 3.57, 95% CI = 1.93–6.62, *p* < 0.0001) were associated with worse DFS (Table 3). Multivariate analysis results showed that advanced FIGO stage (III/IV vs. I/II, HR = 11.61, 95% CI = 2.47–54.60, *p* = 0.002) and high expression of CD44 (+++/++ vs. +/–, HR = 3.45, 95% CI = 1.48–8.04, *p* = 0.004) were independent prognostic factors of poor OS; and advanced FIGO stage (III/IV vs. I/II, HR = 15.53, 95% CI = 3.26–74.12, *p* = 0.001) and high expression of CD44 (+++/++ vs. +/–, HR = 2.45, 95% CI = 1.12–5.37, *p* = 0.025) also independently predicted worse DFS (Table 3).

**Table 3:** Univariate and multivariate analyses of factors associated with overall survival.

**Variables**	**Overall survival**		**Disease-free survival**	
	**HR (95% CI)**	***p*-value**	**HR (95% CI)**	***p*-value**
**Univariate analyses**					
Age (>50 vs. ≤ 50)	0.97 (0.53–1.76)	0.913	0.93 (0.52–1.64)	0.793
Histological subtype (serous vs. non-serous)	1.94 (0.96–3.95)	0.066	1.99 (1.01–3.92)	**0.046**
FIGO stage (III/IV vs. I/II)	9.20 (3.86–21.96)	**<0.0001**	11.36 (4.78–27.03)	**<0.0001**
Histological grade (G1/G2 vs. G3)	1.52 (0.76–3.03)	0.238	1.58 (0.81–3.07)	0.181
Intravascular tumor thrombus (Yes vs. No)	2.08 (1.04–4.16)	**0.039**	1.86 (0.94–3.69)	0.074
CA125 (≥35 U/mL vs. <35 U/mL)	4.62 (1.11–19.26)	**0.035**	4.93 (1.19–20.44)	**0.028**
CD44 (+++/++ vs. +/–)	4.30 (2.20–8.40)	**<0.0001**	3.57 (1.93–6.62)	**<0.0001**
**Multivariate analyses**					
FIGO stage (III/IV vs. I/II)	11.61 (2.47–54.60)	**0.002**	15.53 (3.26–74.12)	**0.001**
CD44 (+++/++ vs. +/–)	3.45 (1.48–8.04)	**0.004**	2.45 (1.12–5.37)	**0.025**

*CA 125, cancer antigen 125; FIGO, Federation of Gynecologists and Obstetricians; HR, hazard ratio; 95% CI, 95% confidence interval. Bold values indicate p-value < 0.05*.

### CD44 Knockdown by Small Silencing RNA Abrogated Both Basal Snail Expression and TGF-β1 Induced Snail Expression in HOPM and HOPM-Snail Cells

**Figure 2:**
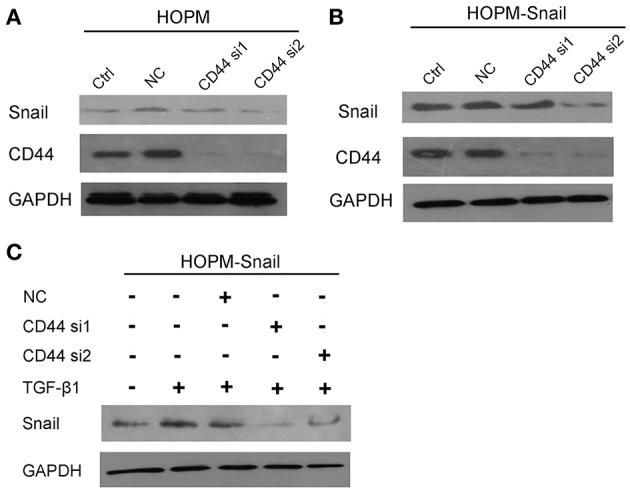
CD44 knockdown abrogated both basal Snail expression and TGF-β1 induced Snail expression. **(A,B)** HOPM and HOPM-Snail cells were transfected with CD44 siRNA 1 and siRNA 2, Snail expression was detected by western blotting analysis. **(C)** HOPM-Snail cells were treated with recombinant TGF-β1 and/or CD44 siRNA, Snail expression was detected by western blotting analysis. A representative blot of triplicate blots was shown. NC, non-specific siRNA control. GAPDH was used as the loading control.

Although HOPM is a highly metastatic ovarian cancer cell line, its endogenous expression of Snail is low, so that it is difficult to observe the effect of CD44 on Snail expression using this cell line. Therefore, Snail high-expression cell line HOPM-Snail was used to observe the regulation of CD44 on Snail expression. Both HOPM and HOPM-Snail cells were transfected with CD44 siRNAs, Snail expression were then detected by western blotting assay. As indicated in Figures 2A,B, both siRNAs effectively suppressed CD44 expression. CD44 siRNAs, especially siRNA2 dramatically abrogated Snail expression in HOPM and HOPM-Snail cells.

As TGF-β1 has been proven to induce Snail expression, we then detected whether CD44 siRNA abrogated TGF-β1-induced Snail expression. As shown in Figure 2C, TGF-β1 promoted Snail expression, which can be dramatically blocked by CD44 siRNAs. Taken together, these data indicated that CD44 may be crucial for the TGF-β1-induced Snail expression.

### CD44 Knockdown Caused a Decrease in ZEB1 and an Increase in Caveolin-1 Expression

**Figure 3:**
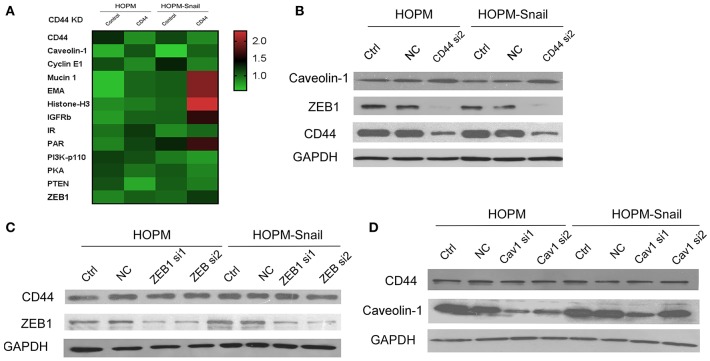
CD44 knockdown caused a decrease in ZEB1 expression and increase in Caveolin-1 expression. **(A)** RPPA analysis of the differential gene expression in HOPM and HOPM-Snail cells transfected with CD44 siRNAs. **(B)** HOPM and HOPM-Snail cells were transfected with CD44 siRNA, the expression of ZEB1 and Caveolin-1 was detected by western blotting analysis. **(C)** HOPM and HOPM-Snail cells were transfected with ZEB1 siRNA, CD44 expression was detected by western blotting analysis. **(D)** HOPM and HOPM-Snail cells were transfected with Caveolin-1 siRNA, CD44 expression was detected by western blotting analysis. A representative blot of triplicate blots was shown. NC, non-specific siRNA control. GAPDH was used as the loading control.

RPPA assay was then performed to elucidate the downstream targets of CD44. These data indicated that Cavolin-1 may be the possible downstream targets of CD44 (Figure 3A). Western blotting data confirmed that CD44 siRNAs caused an increase in Caveolin-1 expression, indicating that CD44 may inhibit the expression of Caveolin-1 (Figure 3B).

Considering the crucial role of Snail in EMT, we hypothesized that CD44 may regulate the EMT process. Next, we examined the effect of CD44 on the other EMT inducers. Our findings showed that CD44 siRNA caused a decrease in ZEB1 expression in HOPM and HOPM-Snail cells, indicating that CD44 may regulate ZEB1 expression (Figure 3B).

We further detected whether ZEB1 exerts a negative feedback regulation on CD44. The HOPM and HOPM-Snail cells were treated with ZEB1 siRNAs, and the expression of CD44 in these cells was detected by western blot analysis. As shown in Figure 3C, ZEB1 siRNAs exerted no effects in CD44 expression, indicating that ZEB1 could not regulate CD44 expression negatively. Similarly, we also detected whether Caveolin-1 modulated CD44 expression (Figure 3C). As shown in Figure 3D, HOPM and HOPM-Snail cells treated with Caveolin-1 siRNAs demonstrated similar CD44 expression compared with their control counterparts, indicating that there is no reciprocal regulation between Caveolin-1 and CD44.

### CD44 Knockdown Resulted in Reduced Cell Migration, Cell Invasion, and Clone Formation in HOPM and HOPM-Snail Cells

**Figure 4:**
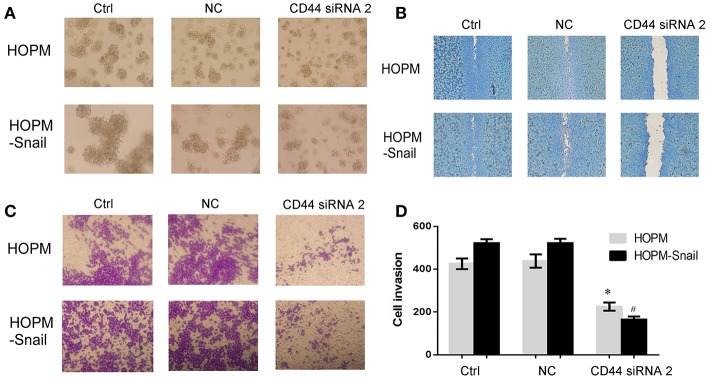
CD44 knockdown resulted in reduced clone formation, cell migration, and cell invasion of HOPM and HOPM-Snail cells. **(A)** HOPM and HOPM-Snail cells were transfected with CD44 siRNA 2, clone formation capacity was detected by 3-D culture assay. **(A)** At 48 h post-transfection, cells in 4% matrigel were seeded in 6-well plate and maintained in 37°C for 7–14 days, formed cell clones were then photographed. A representative image of triplicate experiments was shown. **(B)** HOPM and HOPM-Snail cells were transfected with CD44 siRNA 2, cell migration was detected by wound healing assay. At 48 h post-transfection, a linear wound was generated in the cells with 70–80% confluence and the scratched areas were photographed. A representative image of triplicate experiments was shown. **(C,D)** HOPM and HOPM-Snail cells were transfected with CD44 siRNA 2, cell invasion was detected by transwell assay. At 48 h post-transfection, cells were plated in the upper chamber of an insert and the cells that migrated to the lower surface of the filters were monitored after 48 h incubation. A representative image **(C)** and quantitative analysis **(D)** were shown. *p < 0.05 compared with HOPM control cells. ^#^*p* < 0.05 compared with HOPM-Snail control cells. NC, non-specific siRNA control.

We then observed the effect of CD44 siRNA on the growth, migration and invasion of ovarian cancer. 3D matrigel culture system confirmed that CD44 siRNA abrogated the growth of HOPM and HOPM-Snail cells (Figure 4A). Wound healing assay and transwell showed that CD44 siRNA abrogated cell migration and invasion capacities of HOPM and HOPM-Snail cells (Figures 4B–D). These data indicated that CD44 may be crucial for the growth, migration, and invasion of human ovarian cancer. We also evaluated the expression of CD44 in a series of ovarian cancer cell lines with different invasive potential (Figure S2). CD44 expression in SKOV3 and SKOV3ip cells seemed to be consistent with their invasive capacities as CD44 expression in SKOV3ip is much higher than that in SKOV3 cells. However, CD44 expression in the high metastatic HOPM-Snail cells was not higher than that in HOPM cells. The high metastatic ovarian cancer cell line OVCAR-5 did not even demonstrate CD44 expression. Therefore, although CD44 is crucial for the invasion of ovarian cancer, it may not be the only determinant of invasive capacity of different ovarian cancer cell lines.

## Discussion

In the present study, we demonstrated that CD44 expression positively correlated with FIGO stage and histological grade of ovarian cancer. We observed that up-regulation of CD44 expression was an independent prognostic factor of both poor OS and DFS. Furthermore, our *in vitro* data suggested that CD44 may promote ovarian cancer progression through the EMT process by regulating Snail and ZEB1. Our study suggested that CD44 may serve as a prognostic marker as well as possible treatment target for ovarian cancer.

A number of studies have been conducted to investigate the association between CD44 expression and ovarian cancer prognosis, with controversial results (8–18, 33, 34). Similar to our results, studies reported that ovarian cancer patients with positive expression of CD44 variant had a significantly shorter DFS (8), while low levels of CD44 expression was associated with better survival (12). Another study conducted in the US also reported that the expression of standard CD44 (CD44s) was significantly associated with worse DFS both in univariate (*p* = 0.003) and multivariate (*p* = 0.006) analysis in 56 patients with epithelial ovarian cancer (9). A study from Korea also reported that over expression of CD44s was an independent prognostic factor of lower overall survival rate (17). In addition, expression of CD44 spliced variant 6 (CD44v6) was reported to be associated with a shortened overall survival in stage III-IV patients as well as the recurrence of ovarian serous cancer (10, 11). A more recent meta-analysis pooling data from 2,161 patients further reported that positive expression of CD44 was significantly associated with poor 5-year overall survival (RR = 1.42, 95% CI: 1.01–2.00, *p* = 0.05) (18).

However, there are also studies that did not find any association between CD44 variant expression and survival (13, 33). Some studies also reported that down-regulation of CD44 is associated with unfavorable prognosis (14–16). Loss of CD44s expression was associated with shorter survival, although the association did not maintain statistical significance after multivariate adjustment (15). Some studies showed that expression of CD44 was an independent predictor of favorable 5-year recurrence-free survival (16). More interestingly, there is also research showing that the expression of CD44 splice variant and the survival of ovarian cancer is site dependent (34). Expression of CD44-v10 in metastatic lesions was associated with decreased survival, while expression of CD44s in the primary tumor and the tumor-stroma interface was associated with improved survival (34). These conflicting findings may due to inadequate sample size and/or power, or different splicing variants of CD44.

EMT is a process by which cells gain the ability to escape from the epithelial layer and invade secondary sites, and form metastases (25). Previous data have shown that CD44 may regulate EMT progress by regulating E-cadherin, vimentin, and N-cadherin expression (29). In addition, CD44s was up-regulated upon TGF-β1-induced EMT. Moreover, CD44s over-expression in ovarian cancer cells induced EMT and caused gain of stem-like features and chemoresistance (29). These data indicated that CD44 may regulate the EMT process. On the basis of previous studies, our findings further demonstrated that CD44 siRNAs lead to the decrease of Snail and ZEB1, indicating that CD44 may promote the EMT process through Snail and ZEB1. Snail has been well-characterized as the inducer of EMT and its expression is sufficient for the initiation of EMT (35). ZEB1 is another crucial regulator of EMT. The activation of ZEB1 and Snail has been reported to, either directly or indirectly represses the E-cadherin (CDH1) promoter and drives the EMT process (35, 36). Therefore, our data has elucidated a new mechanism by which CD44 induces the EMT progress.

In addition, our data demonstrated that CD44 knockdown lead to an increase of Caveolin-1. Caveolin-1 is a scaffolding protein which promotes the biogenesis of caveolae (37). It has been reported that Caveolin-1 acts as a tumor suppressor in ovarian and breast cancers, and high expression of Caveolin-1 may correlate with favorable outcome of ovarian cancer (38–40). Presumably, up-regulation of Caveolin-1 in CD44 knockdown ovarian cancer cells may partially contribute to the favorable prognosis of ovarian cancer patients.

Chemotherapy resistance is a main reason of therapeutic failure and poor prognosis of ovarian cancer patients, which becomes a major obstacle of ovarian cancer treatment (5). CD44, as a cancer stem cell marker, also plays a pivotal role in chemoresistance. Recent studies have shown that CD44 knockdown increases the sensitivity to anticancer drugs in breast cancer cells, myeloma, and colon cancer (41–44). Our *in vitro* data further suggested that CD44 silencing caused a decrease in the ZEB1 expression. Notably, down-regulation of ZEB1 resulted in significant inhibition of cisplatin-resistance in ovarian and lung cancers (45, 46). Presumably, CD44 may regulate chemoresistance of ovarian cancer *via* ZEB1. Since we do not have chemoresistance data due to difficulties in clinical follow-up, we could not assess the association between CD44 expression and chemotherapy failure in the present study. However, most of the ovarian cancer patients die due to recurrence after development of chemoresistance. In the future, well-designed cohort studies with a larger sample size and more complete follow-up data, as well as in-depth molecular mechanism researches are needed to elucidate the role of CD44 and ZEB1 in the chemoresistance of ovarian cancer.

Taken together, our present study indicated that CD44 may be pivotal for EMT and closely correlate with the prognosis of ovarian cancer. Clarifying the role of CD44 in the prognosis of ovarian cancer may be of great help for the development of novel treatment strategies.

## Conclusions

In conclusion, our study suggested that high expression of CD44 was correlated with poor prognosis of ovarian cancer patients. Our data also showed that ZEB1, Snail, and Caveolin-1 are regulated by CD44, indicating the crucial role of CD44 in the initiation of EMT in ovarian cancer. Presumably, CD44 may be a potent therapeutic target of human ovarian cancer.

## Data Availability

The datasets generated for this study are available on request to the corresponding author.

## Author Contributions

HZ, YY, and CX conceived and designed study. JZ, BL, and YY performed the cell line studies. YD and HZ wrote the manuscript and assessed the IHC score. YL performed the RPPA analysis. YD reviewed the clinical records and conducted the statistical analysis. All authors read and approved the final manuscript.

### Conflict of Interest Statement

The authors declare that the research was conducted in the absence of any commercial or financial relationships that could be construed as a potential conflict of interest.

## Supplementary Material

Figure S1 CD44 was almost undetectable in normal ovarian tissues. Immunohistochemistry analysis of CD44 expression in four normal ovarian tissues from patients with benign disease who underwent hysterectomy and bilateral appendectomy. Scale bar = 200 μM.

Figure S2 CD44 expression may not necessarily be associated with the invasive potential of different ovarian cancer cell lines. Western blotting analysis of CD44 expression in HOPM, HOPM-Snail, Skov3, Skov3ip, OC316, Hey, ES2, OVCAR-3, OVCAR-5 cell lines. GAPDH was used as the loading control.
