# Supplementary material for: CD44 Expression Predicts Prognosis of Ovarian Cancer Patients Through Promoting Epithelial-Mesenchymal Transition (EMT) by Regulating Snail, ZEB1, and Caveolin-1
Source: Front Oncol. 2019 Aug 21;9:802. doi: 10.3389/fonc.2019.00802 (PMC6712994; doi:10.3389/fonc.2019.00802)
Supplement: Supplementary file 1 [file Table_1.DOC]

**TABLE S1**. The target sequences of small interfering RNAs (siRNAs)

**Gene Target sequences**

CD44 siRNA 1 5’- GCAGATCGATTTGAATATA -3’

CD44 siRNA 2 5’- CCGCTTTGCAGGTGTATTC -3’

ZEB1 siRNA 1   5’- GGCAAGTGTTGGAGAATAA -3’

ZEB1 siRNA 2 5’- CCAGAAATACACAGGGTTA -3’

Caveolin-1 siRNA1 5’- GCATCAACTTGCAGAAAGA -3’

Caveolin-1 siRNA 2 5’- GCAAATACGTAGACTCGGA -3’
