# Supplementary figures and images for: CD44 Expression Predicts Prognosis of Ovarian Cancer Patients Through Promoting Epithelial-Mesenchymal Transition (EMT) by Regulating Snail, ZEB1, and Caveolin-1
Source: Front Oncol. 2019 Aug 21;9:802. doi: 10.3389/fonc.2019.00802 (PMC6712994; doi:10.3389/fonc.2019.00802)

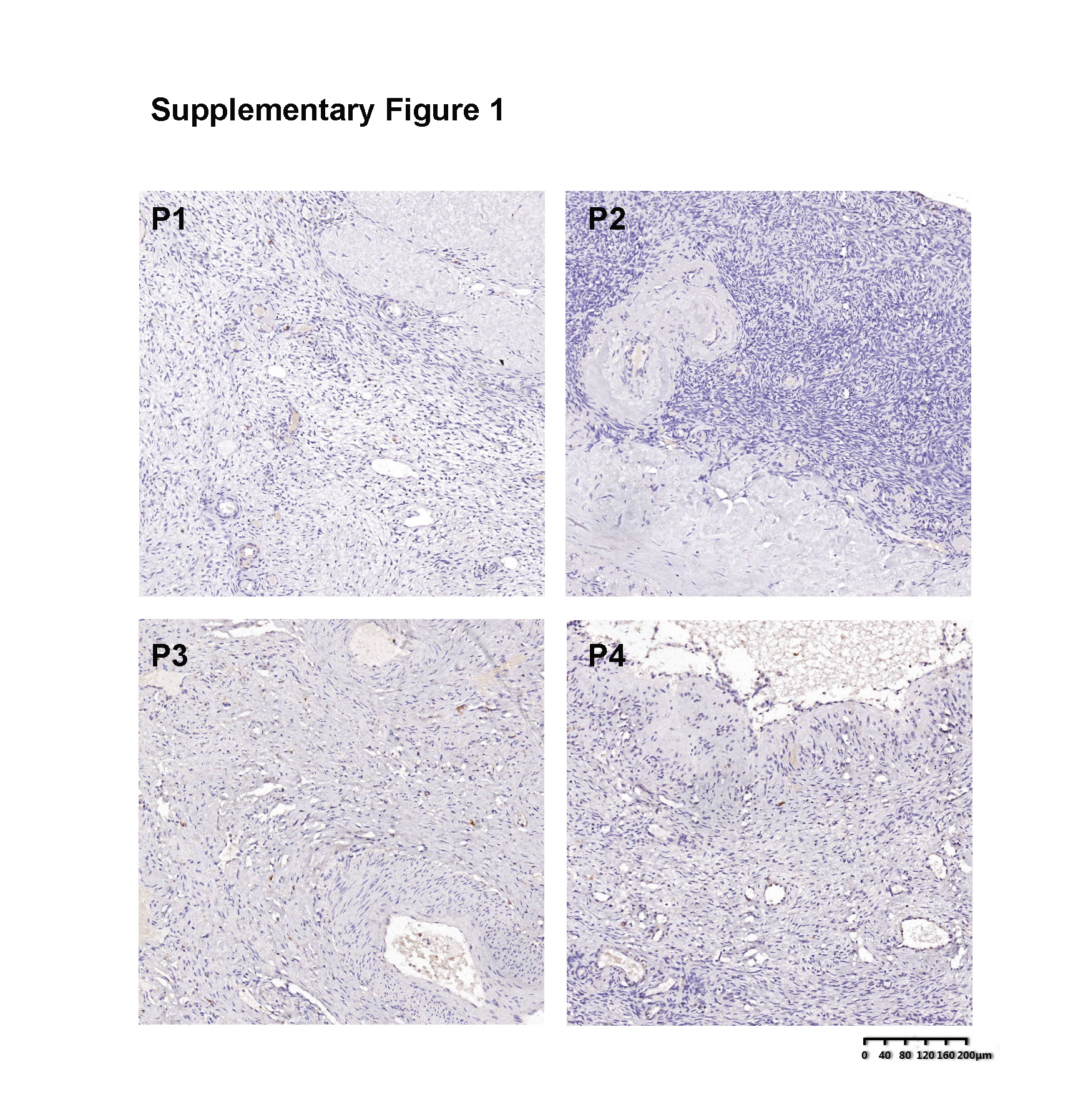

Supplement: Figure S1 — CD44 was almost undetectable in normal ovarian tissues. Immunohistochemistry analysis of CD44 expression in four normal ovarian tissues from patients with benign disease who underwent hysterectomy and bilateral appendectomy. Scale bar = 200 μM. [file Image_1.JPEG]

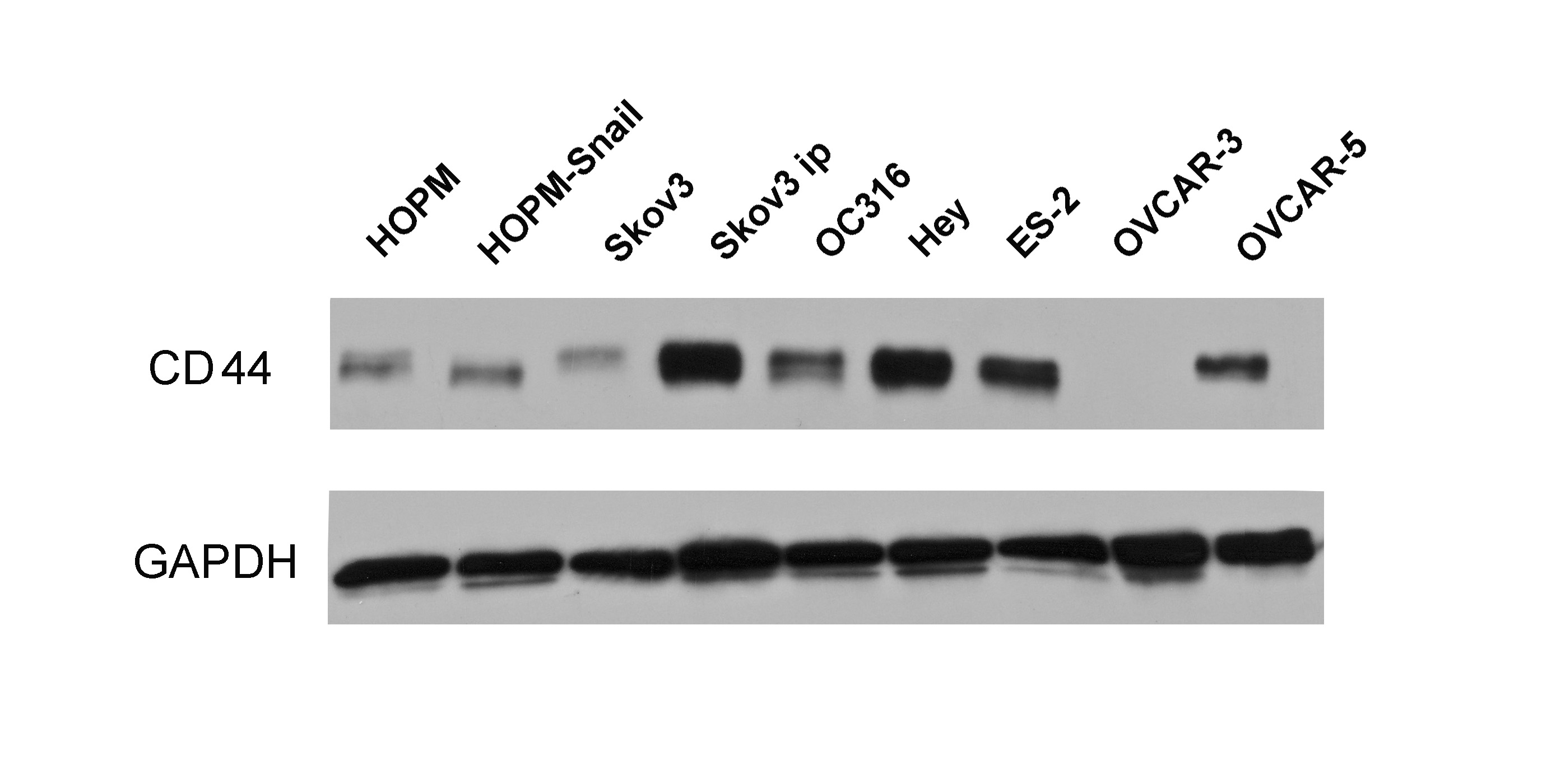

Supplement: Figure S2 — CD44 expression may not necessarily be associated with the invasive potential of different ovarian cancer cell lines. Western blotting analysis of CD44 expression in HOPM, HOPM-Snail, Skov3, Skov3ip, OC316, Hey, ES2, OVCAR-3, OVCAR-5 cell lines. GAPDH was used as the loading control. [file Image_2.JPEG]
